# The functioning of individuals with excessive body weight in the context of the Dark Personality Tetrad and psychological resilience

**DOI:** 10.3389/fpsyt.2025.1674130

**Published:** 2026-01-16

**Authors:** Agnieszka Mateja

**Affiliations:** Institute of Psychology, Humanitas University, Sosnowiec, Poland

**Keywords:** Dark Personality Tetrad, machiavellianism, narcissism, obesity, psychological resiliency, psychopathy, resilience, sadism

## Abstract

**Introduction:**

Obesity is a condition with a complex etiology that is highly prevalent in Western societies. A key factor in understanding the psychological mechanisms underlying obesity and in guiding psychotherapeutic interventions is the identification of psychological factors that influence the ability to regulate body weight. Research on personality correlates reveals significant heterogeneity within this clinical population. Higher body mass is associated with increased levels of neuroticism, decreased extraversion, lower conscientiousness, and greater emotional instability. Furthermore, other studies indicate a positive correlation between obesity and impulsivity, body image dysphoria, perfectionism, disinhibition, and a negative correlation with self-esteem.

**Methods:**

A total of 463 individuals were studied. The group with excessive body weight (BMI > 25) comprised 322 participants, while the group with normal body weight (BMI < 25) included 141 participants. Personality traits associated with the Dark Tetrad (narcissism, psychopathy, machiavellianism, and sadism) were measured using the SD4-PL questionnaire. General psychological resilience, defined as a stable psychological disposition that enables effective adaptation in crises, was assessed using the SPP-25 scale.

**Results:**

No significant differences were found between individuals with excessive body weight (BMI > 25) and those with normal body weight (BMI < 25) in terms of Dark Personality Tetrad traits or psychological resilience. A negative correlation between resilience and sadism was found in the control group. No similar correlation was seen in the study group.

**Conclusions:**

The group of individuals with excessive body weight is heterogeneous in terms of personality traits. There are no differences in the intensity of Dark Tetrad traits (narcissism, psychopathy, machiavellianism, sadism) compared to individuals with normal body weight. Levels of psychological resilience are also similar. Sadism may have different structures and functions in both groups. In people with normal weight, it is personality-related, while in overweight individuals, it is reactive. This alters the connection between sadism and resilience.

## Introduction

### Personality traits and obesity

Obesity is a disease with a complex etiology that is widespread in Western societies (1–3). Identifying psychological factors influencing weight control appears to be a crucial element in understanding the psychological mechanisms involved in obesity and guiding appropriate psychotherapeutic interventions. Research on personality correlates indicates significant heterogeneity within this clinical population (4–7). Differences in personality traits have been observed among individuals with excessive body weight; however, the intensity of these traits does not suggest the presence of personality disorders within this group (8, 9). Higher body mass is associated with increased neuroticism (2, 10–13), reduced extraversion (14), lower levels of conscientiousness (2, 13), and emotional instability (2, 11, 12, 15). Other studies indicate a positive correlation between obesity and impulsivity (13, 16, 17), body image dysphoria, perfectionism, disinhibition, and a negative correlation with self-esteem (3, 18). Higher levels of depression and even diagnosed depressive episodes are observed across the population of individuals with high body weight. However, this is more closely associated with one behavioral aspect of obesity, namely, binge eating (6, 15, 16, 19–28). Episodes of binge eating suggest the possibility of food addiction mechanisms in individuals with excessive body weight. Overeating, particularly in response to negative emotional states, may function as a defensive mechanism that reduces tension and activates the reward system (10, 17, 29–33). Impulsivity and poor self-control observed in individuals with excessive body weight may trigger compulsive behaviors, in this case, compulsive overeating. This self-aggression may result from the suppression of negative affect (3) or avoidance of conflict situations. The occurrence of depression in individuals with excessive body weight may also point to difficulties in expressing anger. Interesting findings show that anger is a significant component in diseases such as hypertension, heart attack, and the accumulation of excessive visceral fat tissue (3). The onset of anger is accompanied by the activation of the autonomic nervous system, as well as sensations of hunger (34).

A study conducted by Imre and Toporak found that people who successfully lost weight were motivated to exceed their own capabilities. In addition, they helped people around them. They attempted not only to overcome their own difficulties but also to support others. The results of studies linking the main dimensions of personality with successful weight loss are inconsistent (35). More detailed research on personality subdimensions is needed. For example, perseverance, defined as remaining determined and steadfast in the face of adversity, increases the effectiveness of bariatric treatment. Persevering individuals will consistently change their lifestyle. In addition, independence could help integrate a new lifestyle into one’s personality. However, no association between independence and weight loss has been observed. It should be noted that the studies were conducted on a relatively small sample. Perhaps too much independence of thought is not conducive to compliance with the requirements of obesity treatment. Social support is also not a factor that aids in treating obesity (36).

### The Dark Personality Tetrad and obesity

The Dark Personality Tetrad encompasses psychological traits associated with antisocial attitudes, such as sadism, psychopathy, narcissism, and machiavellianism. Analyzing the intensity of these traits allows for the identification of tendencies toward antisocial behaviors (37). Individuals suffering from obesity often experience social discrimination due to excessive body weight and are thereby exposed to frequent and prolonged stress (38–40). Vulnerable narcissism is associated with aggressive behaviors, excessive compulsivity, and susceptibility to addiction, particularly alcohol dependence (41–43). Among individuals with excessive body weight, emotional regulation through food is frequently observed, often manifesting as compulsive overeating, which constitutes a form of self-aggression. Impulsivity may lead to behaviors that contribute to weight gain, such as difficulty refraining from eating under stress exposure, or engaging in short-term pleasure-seeking activities (e.g., video games, eating) over those requiring persistence and determination (e.g., physical activity) (12, 17). Obesity disproportionately affects individuals from minority groups or those with low socioeconomic status, which may be linked to higher levels of Dark Tetrad traits. Obesity can also be seen as a consequence of life in an increasingly competitive and stressful society. Vulnerable narcissists, sensitive individuals with a need to feel exceptional, experience shame related to their ambitions, in contrast to classic narcissistic attitudes (44, 45). This leads to an intrapsychic conflict between high expectations and perfectionism (traits often identified among individuals with excessive body weight) and feelings of shame. Such individuals are more susceptible to emotional harm and more sensitive to others’ opinions. This narcissistic stance is associated with high neuroticism, which is also frequently observed in people with excessive body weight (12, 15, 46). These individuals are particularly vulnerable to social stress due to a strong need for external validation, while simultaneously being unable to cope with criticism. They feel judged, excluded, and discriminated against (47–49). Excessive body weight may serve a regulatory function. From a psychological perspective, it may justify an individual’s failure to fulfill internal ambitions, avoid confrontation, and accept a lower-than-expected social status (44, 45). Depression and obesity may be viewed as interrelated responses to an inability to cope with social stress (50).

To date, there is a lack of research specifically addressing the Dark Personality Tetrad in individuals with excessive body weight. Existing studies only report on narcissism (44, 45), though these remain scarce.

### Psychological resilience and obesity

Psychological resilience remains insufficiently explored in the literature on obesity (51, 52). Low resilience plays a detrimental role among individuals undergoing bariatric surgery for obesity. Within the salutogenic model, resilience is a key health-promoting factor (53, 54), enabling adaptive coping in adverse and crises. Individuals with higher resilience more frequently adopt adaptive stress-coping strategies (53–59). Higher levels of psychological resilience in obese individuals who have undergone bariatric surgery are associated with a decrease in episodes of compulsive overeating, reduced symptoms of depression and anxiety, and a lower perceived negative impact of body weight on quality of life (60–63).

Mental resilience is also influenced by self-esteem. Research shows that low self-esteem can lead to obesity, but obesity can also lower self-esteem (64). Low self-esteem is at the root of many mental disorders. There is a bidirectional relationship between obesity and mental health problems (65). On the one hand, obesity can develop due to mental disorders, but it can also contribute to their development. This is primarily related to the stigmatization of people who are overweight (66). This relationship is powerful in the case of depression. The co-occurrence of obesity and depression is associated with a reduced ability to maintain a treatment regimen. This reduces the effectiveness of treatment. Therefore, psychotherapy should be one of the key elements in the treatment of obesity. Research on personality traits coexisting with obesity will enable targeted psychotherapeutic interventions (36).

### The current study

The main objective of this study is to identify the correlation between psychological resilience and antisocial personality traits (sadism, psychopathy, narcissism, and machiavellianism), collectively referred to as the Dark Personality Tetrad, in patients with excessive body weight. The study also evaluated differences between individuals with excessive body weight (BMI > 25) and those with normal body weight (BMI < 25) in terms of both Dark Tetrad traits and psychological resilience.

## Methods

### Participants

A total of 463 individuals participated in the study. The study population was divided into two groups: clinical and control. The clinical group included 322 respondents with excessive body weight (BMI > 25), while the control group consisted of 141 individuals with normal body weight (BMI < 25). BMI was calculated based on self-reported weight and height. Participants in the clinical group were selected from the general population based on their BMI values.

The gender distribution in the group of individuals with excessive body weight is: 41% female and 59% male, and in the group with normal body weight is: 24% female and 76% male.

Individuals with excessive body weight (BMI > 25) were aged between 18 and 70 years (M = 46.19; SD = 13.06). Individuals with normal body weight were aged between 18 and 65 years (M = 36; SD = 11.75).

Sociodemographic and treatment-related data collected via the questionnaire are presented in **Table 1**.

**Table 1:** Sociodemographic and treatment-related data collected via the questionnaire.

Survey data	High-BMI individuals		Normal-BMI individuals		p	V Kramera	Phi
	Number of persons	Percentage (%)	Number of persons	percentage (%)			
EDUCATION					.37	.12	.20
PRIMARY	8	2.45	3	2.04			
MIDDLE	3	.92	2	1.36			
SCHOOL	53	16.21	6	4.08			
VOCATIONAL	97	29.66	57	38.77			
SECONDARY	47	14.37	21	14.28			
BACHELOR’S DEGREE	119	36.39	58	39.45			
MASTER’S DEGREE PLACE OF RESIDENCE VILLAGE	78	23.85	23	15.65	.004	.13	.23
SMALL TOWN	44	13.46	20	13.60			
MEDIUM-SIZED CITY	100	30.58	36	24.49			
LARGE CITY	105	32.11	68	46.26			
MARITAL STATUS					<.001	.46	.46
SINGLE	73	22.32	81	55.10			
MARRIED	193	59.02	47	31.97			
DIVORCED	38	11.62	15	10.20			
WIDOWED	23	7.03	4	2.72			
DO YOU HAVE ANY MEDICAL CONDITIONS?					<.001	.46	.46
YES	145	44.34	137	93.20			
NO	182	55.66	10	6.80			
DO YOU TAKE ANY MEDICATION?					<.001	.27	.47
YES	149	45.57	136	92.52			
NO	178	54.43	11	7.48			

### Procedure

Participants were recruited from the general population. The clinical and control groups were distinguished based on BMI.

Participation in the study was voluntary and anonymous. Participants were informed of this both prior to the study and through a notice included at the beginning of each questionnaire. The study was conducted individually with each participant. Completing the questionnaire, which included sociodemographic data and psychometric instruments, took approximately 15 minutes.

The written questionnaire was handed out in public places like government offices and clinics. Respondents filled out the questionnaire only once. They were informed that they could withdraw from the study at any time.

The study was submitted to and received a favorable recommendation from the Research.

Ethics Committee of the Humanitas University (Opinion no 5/2025; 2025-05-08; Sosnowiec, Poland).

### Measures

The study assessed variables comprising the Dark Personality Tetrad (narcissism, psychopathy, machiavellianism, sadism), as well as the overall level of psychological resilience and its specific traits. Measurements were obtained using the following psychological instruments: the Resilience Measurement Scale (SPP-25) (67). and the Short Dark Tetrad Scale (SD4) (68). Both questionnaires have demonstrated strong psychometric properties. An original survey questionnaire was also used to characterize the clinical and control groups. Within this survey, participants responded to questions regarding gender, age, education, place of residence, marital status, presence of illness, and medication use.

The SD4 measures antisocial personality traits, including machiavellianism, narcissism, psychopathy, and sadism (68). SD4-PL is the Polish adaptation of the Short Dark Tetrad (SD4) questionnaire developed by D.L. Paulhus, E.E. Buckels, P.D. Trapnell, and D.N. Jones. The Polish version was adapted by P. Debski, P. Palczynski, M. Garczarczyk, M. Piankowska, K. Haratyk, and M. Meisner (69). The Polish version of SD4 is currently undergoing psychometric testing. The SD4-PL version was obtained from the authors of the study. The SD4-PL scale consists of 28 statements rated on a 5-point Likert scale, where 1 indicates Strongly disagree and 5 indicates Strongly agree. It is a newly developed scale, methodologically constructed during the work on the adaptation article. Cronbach’s alpha coefficients in the validation study: psychopathy is.71, narcissism is.79, Machiavellianism is.68, sadism is 77. Cronbach’s alpha coefficient of the entire scale in the current study is.68.

The SPP-25 was developed by Zygfryd Juczynski and Nina Oginska-Bulik (67). It demonstrates good psychometric properties. Cronbach’s alpha in the current study is 0.89, the standard error of measurement for the overall score is 3.81, and the reliability of individual subscales ranges from 0.67 to 0.75, which is considered satisfactory. The SPP-25 scale consists of 25 statements rated on a 5-point Likert scale, where 0 indicates Definitely Not and 4 indicates Definitely Yes. The scale measures aspects of psychological resilience understood as personality traits. It can be used to assess personality-based predispositions in individuals, particularly exposed to stress. The tool is based on self-report. The SPP-25 provides a general resilience score as well as levels of the following five resilience components:

1. Perseverance and determination in action.
2. Openness to new experiences and sense of humor.
3. Personal competence in coping and tolerance of negative emotions.
4. Tolerance of failure and perceiving life as a challenge.
5. Optimistic outlook on life and the ability to mobilize oneself in difficult situations (67).

### Data analysis plan

The study compared the overall resilience index, and individual resilience factors in the groups in terms of Dark Tetrad personality traits.

The following research hypotheses were formulated:

H1: People who are overweight have higher Dark Tetrad scores than people of normal weight.
H2: The level of narcissism is higher in overweight individuals compared to those of normal weight.
H3: People who are overweight have lower levels of mental resilience.
H4: The correlation between narcissism and psychological resilience is higher in people who are overweight.

The normality of the data distribution was evaluated using the Shapiro-Wilk and Kolmogorov-Smirnov tests.

A Mann- Whitney U test was used to compare the clinical and control groups. The analysis was supplemented by the interpretation of effect size values using eta- squared. The next step involved conducting a correlation analysis within the overweight group between personality traits encompassed by the Dark Tetrad framework and both the overall resilience score and factor-specific resilience scores. The correlations between variables were examined using Spearman’s correlation (70).

The data from this study are not publicly available due to the sensitive nature of the studied population.

## Results

### Descriptive statistics

The mean scores and standard deviations for the Resilience (SPP-25), and Dark Tetrad (SD4) are presented in **Tables 2**, **3**.

**Table 2:** Descriptive statistics for resilience.

Psychological Resilience (SPP-25)	M	SD	𝛼3	SE	𝛾2	SE	W	p	D	p
Sum score	70.61	15.28	-.55	.11	.7	.23	.98	<.001	.05	.006
1. Perseverance and determination in action	14.33	3.85	.93	.11	11.84	.23	.9	<.001	.11	<.001
2. Openness to new experience and sense of humor	15.33	3.15	-.88	.11	1.59	.23	.94	<.001	.11	<.001
3. Personal competence to cope with and tolerance of negative emotions	13.82	3.67	-.57	.11	.32	.23	.97	<.001	.09	<.001
4. Tolerance for failures and treatment of life as challenges	14.27	3.39	-.85	.11	1.59	.23	.95	<.001	.11	<.001
5. Optimistic attitude to life and the ability to mobilize in difficult situations	12.85	3.94	-.44	.11	.16	.23	.98	<.001	.08	<.001

**Table 3:** Descriptive statistics for Dark Tetrad.

Dark Tetrad (SD4)	M	SD	𝛼3	SE	𝛾2	SE	W	p	D	p
Narcissism	18.51	5.81	.28	.11	-.06	.23	.99	<.001	.06	<.001
Psychopathy	14.03	5.15	.97	.11	1	.23	.93	<.001	.11	<.001
Machiavellianism	21.72	5.07	-.40	.11	1.16	.23	.98	<.001	.07	<.001
Sadism	14.34	5.89	.81	.11	.22	.23	.93	<.001	.1	<.001

### Group differences in the psychological resilience and Dark Personality Tetrad traits between the High- and Normal-BMI groups

To verify the differences between high-BMI (>25.00) and normal-BMI (<25.00) participants in resilience and Dark Tetrad traits, we conducted a series of Mann- Whitney U tests. For detailed results, see **Table 4**.

**Table 4:** Mean comparisons for psychological resilience and Dark Tetrad between groups.

Dependent variable	High BMI (*n* = 322)		Normal BMI (*n* = 141)		Mann-Whitney U test		
	*M*	*SD*	*M*	*SD*	*Z*	*p*	η²
Psychological Resilience							
Sum score	70.20	15.52	71.48	14.75	-1.43	.15	<0,00
1. Perseverance and determination in action	14.32	3.98	14.35	3.56	-.86	.39	<0,00
2. Openness to new experience and sense of humor	15.27	3.17	15.46	3.12	-.78	.43	<0,00
3. Personal competence to cope with and tolerance of negative emotions	13.68	3.75	14.12	3.47	-1.61	.11	<0,00
4. Tolerance for failures and treatment of life as challenges	14.11	3.56	14.62	2.98	-1.55	.12	<0,00
5. Optimistic attitude to life and the ability to mobilize in difficult situations	12.82	3.87	12.92	4.10	-.63	.53	<0,00
Dark Tetrad							
Narcissism	18.18	5.66	19.23	6.09	-1.67	.09	<0,00
Psychopathy	14.11	5.25	13.83	4.96	-.34	.74	<0,00
Machiavellianism	21.89	4.88	21.36	5.48	-.99	.32	<0,00
Sadism	14.12	5.72	14.81	6.25	-.89	.37	<0,00

The obtained data show that participants with high BMI (> 25.00) are not, on average, more psychologically resilient than those with normal BMI (< 25.00). In other words, body mass index does not appear to be associated with overall levels of psychological resilience in our sample. Furthermore, we did not observe any statistically significant differences between the two BMI groups in any of the Dark Tetrad personality traits, including narcissism, psychopathy, machiavellianism, and sadism. These findings suggest that higher body mass is not linked to elevated levels of dark personality traits or to reduced psychological resilience.

### The relation between psychological resilience and Dark Personality Tetrad for High-BMI people

To examine the relationship between various dimensions of psychological resilience and dark personality traits among individuals with high BMI (>25.00), we conducted a correlation analysis using Spearman’s correlation. The results are presented in **Table 5**. This analysis allowed us to explore potential patterns of association between psychological strengths and maladaptive personality characteristics within this subgroup.

**Table 5:** Correlation between psychological resilience and Dark Tetrad (High-BMI group).

Psychological Resilience	Dark Tetrad			
	Narcissism	Psychopathy	Machiavellianism	Sadism
Sum score	.39^**^	.04	.09	.04
1. Perseverance and determination in action	.22^**^	-.04	.06	-.00
2. Openness to new experience and sense of humor	.32^**^	.04	.07	.05
3. Personal competence to cope with and tolerance of negative emotions	.40^**^	.07	.09	.09
4. Tolerance for failures and treatment of life as challenges	.36^**^	.03	.08	.02
5. Optimistic attitude to life and the ability to mobilize in difficult situations	.34^**^	.02	.06	.00

^**^p <.01.

The results indicate that among participants with high BMI, there is a robust positive relationship between narcissism and psychological resilience, including its various subdimensions. This suggests that individuals with higher levels of narcissism may also perceive themselves as more resilient in the face of adversity.

### The relation between psychological resilience and Dark Personality Tetrad traits in the High- and Normal-BMI groups

We conducted a Spearman’s *r* correlation analysis to investigate how psychological resilience and Dark Triad personality traits are related to BMI. In addition to the correlations computed for the entire sample, we also calculated correlations separately for participants with high BMI and those with normal BMI. The results are presented in **Table 6**.

**Table 6:** Correlation between BMI, resilience and Dark Tetrad for High- and Normal-BMI participants.

Variables	Group		
	High-BMI	Normal-BMI	All
Psychological Resilience			
Sum score	-.06	-.03	-.09
1. Perseverance and determination in action	-.06	-.06	-.07
2. Openness to new experience and sense of humor	-.09	.05	-.07
3. Personal competence to cope with and tolerance of negative emotions	.01	-.03	-.06
4. Tolerance for failures and treatment of life as challenges	-.04	.00	-.08
5. Optimistic attitude to life and the ability to mobilize in difficult situations	-.08	-.09	-.07
Dark Tetrad			
Narcissism	-.04	-.1	-.09^*^
Psychopathy	-.03	.06	.01
Machiavellianism	-.08	-.02	.00
Sadism	-.05	.09	-.05

^*^p <.05.

The obtained result suggest that in most cases, BMI does not relate to psychological resilience, as well as most of the Dark Triad personality traits. We did not observe significant results for the sample split to High- and Normal-BMI groups. However, when analyzing the whole sample, we observe a significant negative relation between BMI and narcissism. This means that with the increase of BMI, the level of narcissism slightly decreases.

## Discussion

The assessment of Dark Tetrad personality traits (narcissism, psychopathy, machiavellianism, and sadism) did not reveal any significant differences between the clinical group (BMI > 25) and the control group (BMI < 25). A review of studies examining personality traits in individuals with excessive body weight indicates that this population is heterogeneous in terms of personality characteristics (5). It is likely that individuals with excessive body weight exhibit elevated levels of certain personality traits; however, these levels are not markedly increased, nor are they indicative of a predisposition toward the development of personality disorders. The traits encompassed by the Dark Tetrad approximate the diagnostic criteria for personality disorders, which may explain the absence of elevated levels of these traits in individuals with excessive body weight. Personality traits that have been commonly identified in individuals with excessive body weight include high levels of neuroticism (2, 10, 12–15), low levels of extraversion (14, 15), low levels of conscientiousness (11, 13, 15), and high levels of impulsivity (12, 13, 16, 17).

Dark Tetrad personality traits often develop among minority groups that experience discrimination and deprivation of needs. In this context, refugee and migrant populations are frequently discussed (71, 72). Individuals with excessive body weight also constitute a group that experiences discrimination (38–40); however, unlike migrants, they have not changed their cultural or environmental setting. Therefore, they do not perceive themselves as alien within their environment and are not compelled to develop antisocial defense mechanisms typical of Dark Tetrad traits. It is likely that they have access to greater social support compared to refugees. This hypothesis requires further detailed investigation. Additionally, we do not possess information on the duration of participants’ obesity, which limits our understanding of the extent to which they have been exposed to discrimination over time.

Similarly, individuals in the clinical group (BMI > 25) did not differ from those in the control group (BMI < 25) in terms of psychological resilience, across any of its dimensions. There is a lack of research addressing psychological resilience in individuals with excessive body weight. Existing studies primarily focus on patients seeking treatment, particularly those undergoing bariatric surgery. These samples are predominantly composed of women (60–63). An improvement in psychological resilience has been observed among individuals who achieved weight reduction following bariatric surgery (51, 52, 63). Individuals who opt for surgical intervention may represent a distinct subgroup within the obese population (73). Undergoing bariatric surgery requires preliminary weight loss and dietary changes, which suggests that these individuals are determined, motivated, and capable of initiating a demanding lifestyle transformation. In the present study, individuals with excessive body weight were recruited from the general population based solely on their BMI index and were not seeking treatment for obesity.

Narcissism is the only factor that negatively correlates with BMI in the entire population. This correlation is very weak. No such correlation was observed among people with high BMI. It is likely that the relationship between BMI and narcissism is not linear, and that among healthy people with low BMI, narcissism increases slightly faster. Due to the weak correlation and the lack of a logical explanation for this relationship, further studies are needed.

A negative correlation between resilience and sadism was found in the control group. No similar correlation was seen in the study group. Resilience likely plays different roles in both groups. In the group of people with normal body weight, higher resilience is linked to lower levels of sadism. This may suggest its protective role against antisocial traits. In this group, sadism is more personality-driven, and resilience encourages the development of adaptive coping strategies, decreases the need to regulate tension through sadistic tendencies, and is related to empathy, impulse control, and effective emotion regulation. The absence of a similar correlation in the group of overweight individuals may suggest that resilience in this group mainly helps them adapt to chronic stress and stigmatization rather than reducing hostile tendencies. Resilience in the study group appears to focus more on coping with suffering than on regulating aggression, meaning it is more about survival and less about social interaction. Consequently, the link between resilience and sadism in the clinical group may be weak or nonexistent. The asymmetric pattern of results suggests that resilience is not a universal protective factor against dark traits, but its importance varies depending on the psychosocial context. These findings may serve as a foundation for future moderation analyses, as the groups in the current study are diverse.

In this study, overweight individuals were selected from the general population to avoid overrepresentation of individuals seeking bariatric treatment in the study group. Studies on obesity should focus on different groups of overweight individuals: those seeking treatment and those not seeking treatment, those with binge eating disorder, and those with a precise mechanism of food addiction that regulates emotions. Focusing research only on individuals seeking surgical treatment may distort the results, as these are individuals with specific personality traits who are better at coping with crises.

Despite conflicting results, there appear to be associations between personality traits and the effect of obesity treatment. Discovering these associations may help in the psychological treatment of overweight patients. Larger-scale studies are needed.

### Limitations

The clinical group in this study was not stratified based on whether participants were undergoing treatment for obesity. Additionally, the tendency toward compulsive overeating was not assessed, despite evidence suggesting that individuals with such tendencies are particularly predisposed to mental health disorders, especially depressive episodes.

BMI measurements were based on respondents’ reported data. This reduces the data’s reliability. Future studies should be based on objective measurements of body weight and height.

In the current study, there is an imbalance in the study and control groups in terms of gender and age. In subsequent studies, this imbalance can be accounted for by controlling for these variables statistically. It would be advisable to use covariance analysis or regression analysis.

The study did not account for respondents’ socio-economic status. This aspect may influence both the development of obesity and the development of personality traits.

Recruiting study participants using the snowball method significantly limits the representativeness of the sample. Among other things, this is due to the greater number of highly motivated participants and the higher percentage of respondents who maintain social contacts. This method of recruiting participants was chosen because it allows for the selection of a research group from the general population. In this way, the research group is not dominated by individuals seeking bariatric treatment.

The study is cross-sectional, so it is impossible to draw causal conclusions.

## Conclusions

The population of individuals with excessive body weight is heterogeneous in terms of personality traits. No differences were found between this group and individuals with normal body weight regarding the intensity of Dark Tetrad personality traits (narcissism, psychopathy, machiavellianism, sadism). A negative correlation between resilience and sadism was found in the control group. No similar correlation was seen in the study group. Sadism may have different structures and functions in both groups. In people with normal weight, it is personality-related, while in overweight individuals, it is reactive. This alters the connection between sadism and resilience.

There remains a lack of research on personality traits among obese individuals who are not seeking obesity treatment. It may be worthwhile to compare treatment-seeking and non-treatment-seeking subgroups within the obese population. Furthermore, it is important to consider the duration of obesity, as long-term exposure to stress may influence personality functioning.

## Data Availability

The raw data supporting the conclusions of this article will be made available by the authors, without undue reservation.
