# Supplementary material for: The functioning of individuals with excessive body weight in the context of the Dark Personality Tetrad and psychological resilience
Source: Front Psychiatry. 2026 Jan 16;16:1674130. doi: 10.3389/fpsyt.2025.1674130 (PMC12855447; doi:10.3389/fpsyt.2025.1674130)
Supplement: Supplementary file 1 [file Table1.docx]

**SUPPLEMENTARY MATERIALS**

**The functioning of individuals with excessive body weight in the context of the Dark Personality Tetrad and psychological resilience**

**Table S1. Descriptive statistics for resilience (High-BMI group).**

| **Psychological Resilience (SPP-25)** | **M** | **SD** | **𝛼3** | **SE** | **𝛾2** | **SE** | **W** | **p** | **D** | **p** |
| --- | --- | --- | --- | --- | --- | --- | --- | --- | --- | --- |
| Sum score | 70.2 | 15.52 | -.47 | .14 | .74 | .27 | .98 | <.00 | .05 | .08 |
| 1. Perseverance and determination in action | 14.32 | 3.98 | 1.48 | .14 | 15.22 | .27 | .88 | <.00 | .1 | <.00 |
| 1. Openness to new experience and sense of humor | 15.27 | 3.17 | -.8 | .14 | 1.38 | .27 | .95 | <.00 | .09 | <.00 |
| 1. Personal competence to cope with and tolerance of negative emotions | 13.68 | 3.75 | -.52 | .14 | .31 | .27 | .97 | <.00 | .08 | <.00 |
| 1. Tolerance for failures and treatment of life as challenges | 14.11 | 3.56 | -.87 | .14 | 1.66 | .27 | .95 | <.00 | .11 | <.00 |
| 1. Optimistic attitude to life and the ability to mobilize in difficult situations | 12.82 | 3.87 | -.39 | .14 | .09 | .27 | .98 | <.00 | .08 | <.00 |

**Table S2. Descriptive statistics for resilience (Normal-BMI group).**

| **Psychological Resilience (SPP-25)** | **M** | **SD** | **𝛼3** | **SE** | **𝛾2** | **SE** | **W** | **p** | **D** | **p** |
| --- | --- | --- | --- | --- | --- | --- | --- | --- | --- | --- |
| Sum score | 71.72 | 14.77 | -.82 | .2 | .8 | .4 | .96 | <.00 | .1 | .00 |
| 1. Perseverance and determination in action | 14.42 | 3.56 | -.78 | .2 | .22 | .4 | .94 | <.00 | .15 | <.00 |
| 1. Openness to new experience and sense of humor | 15.46 | 3.15 | -1.09 | .2 | 2.28 | .4 | .93 | <.00 | .14 | <.00 |
| 1. Personal competence to cope with and tolerance of negative emotions | 14.21 | 3.41 | -.7 | .2 | .51 | .4 | .96 | <.00 | .12 | <.00 |
| 1. Tolerance for failures and treatment of life as challenges | 14.66 | 3.02 | -.69 | .2 | .45 | .4 | .96 | <.00 | .12 | <.00 |
| 1. Optimistic attitude to life and the ability to mobilize in difficult situations | 12.97 | 4.16 | -.56 | .2 | .31 | .4 | .97 | <.00 | .11 | <.00 |

**Table S3. Descriptive statistics for Dark Tetrad (High-BMI group).**

| **Dark Tetrad (SD4)** | **M** | **SD** | **𝛼3** | **SE** | **𝛾2** | **SE** | **W** | **p** | **D** | **p** |
| --- | --- | --- | --- | --- | --- | --- | --- | --- | --- | --- |
| Narcissism | 18.18 | 5.66 | .25 | .14 | -.00 | .27 | .99 | .00 | .06 | .00 |
| Psychopathy | 14.11 | 5.25 | 1 | .14 | 1.05 | .27 | .93 | <.00 | .12 | <.00 |
| Machiavellianism | 21.89 | 4.88 | -.36 | .14 | 1.49 | .27 | .98 | <.00 | .07 | <.00 |
| Sadism | 14.12 | 5.72 | .83 | .14 | .4 | .27 | .94 | <.00 | .1 | <.00 |

**Table S4. Descriptive statistics for Dark Tetrad (Normal-BMI group).**

| **Dark Tetrad (SD4)** | **M** | **SD** | **𝛼3** | **SE** | **𝛾2** | **SE** | **W** | **p** | **D** | **p** |
| --- | --- | --- | --- | --- | --- | --- | --- | --- | --- | --- |
| Narcissism | 19.26 | 6.12 | .32 | .2 | -.21 | .41 | .98 | .04 | .06 | .2 |
| Psychopathy | 13.87 | 5.02 | .9 | .2 | .84 | .41 | .94 | <.00 | .12 | <.00 |
| Machiavellianism | 21.25 | 5.55 | -.4 | .2 | .56 | .41 | .98 | .05 | .09 | .00 |
| Sadism | 14.85 | 6.36 | .76 | .2 | -.18 | .41 | .93 | <.00 | .11 | <.00 |

**Table S5. Correlation between psychological resilience and Dark Tetrad (Normal-BMI group).**

|  | **Dark Tetrad** | | | |
| --- | --- | --- | --- | --- |
|  | Narcissism | Psychopathy | Machiavellianism | Sadism |
| **Psychological Resilience** |  |  |  |  |
|  |  |  |  |  |
| Sum score | **.33**** | -.11 | .11 | **-.21*** |
|  |  |  |  |  |
| 1. Perseverance and determination in action | **.28**** | -.16 | .16 | **-.26**** |
|  |  |  |  |  |
| 1. Openness to new experience and sense of humor | **.30**** | -.1 | .08 | **-.17*** |
|  |  |  |  |  |
| 1. Personal competence to cope with and tolerance of negative emotions | **.27**** | -.08 | -.04 | **-.19*** |
|  |  |  |  |  |
| 1. Tolerance for failures and treatment of life as challenges | **.28**** | -.17 | .06 | **-.19*** |
|  |  |  |  |  |
| 1. Optimistic attitude to life and the ability to mobilize in difficult situations | **.31**** | .03 | .15 | -.11 |

*Note.* ^**^*p* <.01, *^*^p* <.05.
